# Abrogated expression of DEC1 during oesophageal squamous cell carcinoma progression is age- and family history-related and significantly associated with lymph node metastasis

**DOI:** 10.1038/bjc.2011.25

**Published:** 2011-02-15

**Authors:** V C L Wong, J M Y Ko, R Z Qi, P J Li, L D Wang, J-L Li, Y P Chan, K W Chan, E J Stanbridge, M L Lung

**Affiliations:** 1 Department of Clinical Oncology and Center for Cancer Research, University of Hong Kong, Room L2-23, 2/F, Laboratory Block, 21 Sassoon Road, Pokfulam, Hong Kong (SAR) HKSAR, People's Republic of China; 2 Division of Life Science, Hong Kong University of Science and Technology, Hong Kong (SAR), People's Republic of China; 3 Department of Pathology, Henan Key Laboratory for Esophageal Cancer, Laboratory for Cancer Research, Experimental Center for Medicine, Zhengzhou University, Zhengzhou, Henan, People's Republic of China; 4 Department of Pathology, Yaocun Esophageal Cancer Hospital, Linzhou, Henan, People's Republic of China; 5 Department of Pathology, University of Hong Kong, Hong Kong (SAR), People's Republic of China; 6 Department of Microbiology and Molecular Genetics, University of California, Irvine, CA, USA

**Keywords:** deleted in oesophageal cancer 1 (DEC1), oesophageal squamous cell carcinoma, cancer progression, oesophageal cancer family history, subcellular localisation

## Abstract

**Background:**

Oesophageal squamous cell carcinoma (SCC) causes the highest number of cancer deaths in some regions of Northern China. Previously, we narrowed down a critical region at 9q33-34, identified to be associated with tumour-suppressive function of *deleted in oesophageal cancer 1* (*DEC1*) in oesophageal SCC.

**Methods:**

We generated DEC1 antibody and constructed tissue microarrays (TMAs) utilising tissue specimens from Henan, a high-risk region for oesophageal SCC, to investigate the importance of DEC1 expression in this cancer.

**Results:**

Tissue microarray immunohistochemical staining reveals significant loss of DEC1 from hyperplasia, to tumour, and to lymph node metastasis. In addition, the loss of DEC1 in tumour is age-dependent. Interestingly, there is significant abrogation of DEC1 expression in patients with a family history of oesophageal SCC. Deleted in oesophageal cancer 1 localises to both the cytoplasm and nucleus. The vesicular pattern of DEC1 in the cytoplasm appears to localise at the Golgi and Golgi–endoplasmic reticulum intermediate compartment.

**Conclusion:**

This is the first TMA study to suggest a clinical association of DEC1 in lymph node metastatic oesophageal SCC, early onset oesophageal SCC and familial oesophageal SCC development. Subcellular localisation of DEC1 and its expression in oesophageal SCC tissues provide important insight for further deciphering the molecular mechanism of DEC1 in oesophageal SCC development.

Oesophageal carcinoma occurs with an especially high frequency in certain regions of Northern China, including the high-risk region of Henan (Li, 1982). Allelic losses on human chromosomes hallmark the presence of tumour-suppressor genes involved in cancer development. Frequent loss of heterozygosity (LOH) on chromosome 9 is reported in squamous cell carcinoma (SCC) of the lung and of the head and neck (Tsuchiya *et al*, 1992). In oesophageal SCC, this chromosome 9 loss is also commonly observed (Ko *et al*, 2001). Previously, we identified a critical region at 9q33-34 in oesophageal SCC and identified the importance of *deleted in oesophageal cancer 1* (*DEC1*), which suppresses anchorage-independent growth *in vitro* and tumourigenesis *in vivo* (Nishiwaki *et al*, 2000; Yang *et al*, 2005). Reverse transcriptase–PCR analysis shows its frequent downregulation in an oesophageal SCC cell line panel and primary tumours, indicating that DEC1 may serve as an early detection biomarker for oesophageal cancer patients (Leung *et al*, 2008). Despite the tumour-suppressive role of DEC1, there are as yet no studies reported regarding either the role of the protein or its clinical relevance in oesophageal SCC development. A polyclonal antibody was generated against DEC1 and used to analyse a tissue microarray (TMA) constructed with tissues from the oesophageal SCC high-risk region of Henan, China. Immunohistochemical (IHC) staining was used to study the association of DEC1 expression according to status of cancer progression, age, and association with familial oesophageal cancer. Finally, to better understand the molecular mechanism for DEC1 function, we examined its subcellular protein cytolocalisation.

## Materials and methods

### Cell lines, culture conditions, and clinical specimens

The pcDNA 3.1(+)/*DEC1* transfectants and pcDNA3.1(+) vector-alone control were cultured in growth medium containing 400 *μ*g ml^–1^ G418 (CalBiochem, San Diego, CA, USA), as previously described (Leung *et al*, 2008). The SLMT-1 cell line was established from a Hong Kong Chinese oesophageal SCC patient (Tang *et al*, 2001). An immortalised normal oesophageal epithelial cell line (NE1) was cultured in keratinocyte serum-free medium (Invitrogen, Carlsbad, CA, USA), as previously described (Zhang *et al*, 2006). Oesophageal SCC patient tissues were collected from the Yaocun Esophageal Cancer Hospital, Linzhou, Henan, China from 2005 to 2007.

### Antibody generation and purification

Bacterial His-tag and GST-tag protein purification systems were used to purify the DEC1 protein. The *DEC1* 210 bp cDNA fragment was subcloned from pCR3.1-DEC1 into pGEX-4T-1 (Amersham Pharmacia Biotech, Uppsala, Sweden) at *Eco*RI and *Sal*I restriction sites and into pET28a (Novagen, Madison, WI, USA) at *Eco*RI and *Xho*I sites. Ni-NTA agarose (Qiagen, Hilden, Germany) was used to purify His-tagged DEC1 protein according to the manufacturer's protocol. Purified proteins were dialysed and then emulsified in adjuvant (Sigma-Aldrich, St Louis, MO, USA). Subsequently, it was used to immunise New Zealand 2-month-old white rabbits by subcutaneous methods. Glutathione agarose (GE Healthcare, London, Canada) was used to purify GST-DEC1 protein according to the manufacturer's protocol. The purified GST-tagged DEC1 protein was transferred to a nitrocellulose membrane by SDS–PAGE. The membrane region containing the GST-tagged DEC1-sized protein was cut out and incubated with 10 ml of the rabbit serum at 4 °C overnight. Then 0.1 M glycine buffer (pH 2.0) was added to elute the antibody. One-tenth volume of Tris-Cl buffer (pH 8.5) was then added to neutralise the glycine buffer.

### Western blot analysis

Preparation of cell lysates, protein electrophoresis, and transfer were as previously described (Wong *et al*, 2008). Primary antibody incubation was performed with DEC1 polyclonal antibody (1 : 1000). Ab-1 antibody (1 : 10 000; Calbiochem, Darmstadt, Germany) was used for detecting alpha-tubulin as a loading control.

### TMA and IHC staining

A TMA was constructed using tissues of 196 oesophageal SCC patients and 32 non-oesophageal SCC patients from Henan provided by LD Wang. It comprises normal epithelium from 32 non-oesophageal SCC individuals and 35 matched normal-appearing oesophageal epithelia, 150 hyperplastic epithelia, 71 carcinoma *in situ*, 184 primary tumours, and 28 lymph node metastatic tumours from oesophageal SCC patients. Table 1 shows the clinical information of these patients. The family history-positive (FH+) cases were from individuals having two members or more with oesophageal SCC within three consecutive generations. Immunohistochemical staining was performed as previously described (Yuen *et al*, 2007) using DEC1 polyclonal antibody (1 : 100 dilution) as the primary antibody. The stained sections were examined by a pathologist (KW Chan), who had no previous knowledge of the clinico–pathological data of the patients. The intensity of staining was graded by an arbitrary scale that ranged from 0 to 3, representing negative (‘0’), weak (‘1’), moderate (‘2’), and strong (‘3’) staining, respectively. Staining values of 0 and 1 were classified as low expression, while 2 and 3 were classified as high expression.

### Statistical analysis

Associations between clinical pathological information of oesophageal SCC patients and expression of DEC1 were analysed by *χ*^2^ test using SPSS. A *P*-value below 0.005 was considered statistically significant after Bonferroni adjustment to control for type I error from multiple comparisons (Figure 3C). The trend test for measuring the correlation of DEC1 expression status (high and low DEC1 staining) with ESCC progression states that include normal, hyperplasia, carcinoma *in situ*, tumour, and lymph node metastasis, was carried out by Gamma test. A *P*-value of <0.05 was considered statistically significant.

### Subcellular fractionation

Subcellular fractionation was performed as previously described (Guillemin *et al*, 2005). Monolayer cultures of NE1 cells on 100 mm diameter culture dishes were harvested by trypsinisation and resuspended for 5 min on ice in 500 *μ*l extraction buffer (10 mM HEPES, 10 mM NaCl, 1 mM KH_2_PO_4_, 5 mM NaHCO_3_, 5 mM EDTA, 1 mM CaCl_2_, and 0.5 mM MgCl_2_). A volume of 50 ml of 2.5 M sucrose was added to restore isotonic conditions. The lysate was centrifuged at 6300 **g** for 5 min. The supernatant was kept as the cytoplasmic fraction. The pellet was resuspended in 1 ml 500 *μ*l extraction buffer with 2.5 M sucrose and then centrifuged again at 6300 **g** for 5 min. The pellet was considered the nuclear fraction. Anti-lamin A antibody (1 : 1000, Abcam, Cambridge, UK) was utilised to detect the nuclear fraction (1 : 100 dilution).

### Immunostaining and colocalisation study

NE1 cells were grown until 80% confluence in 35 mm culture dishes on coverslips. Cells were then fixed in 4% paraformaldehyde for 10 min at room temperature and permeabilised with 1% Triton X-100 in PBS for 10 min at room temperature. The procedure for immunostaining was the same as previously described (Leung *et al*, 2008). A nuclear stain, DAPI (Sigma-Aldrich), was applied at a concentration of 0.1 mg ml^–1^. Finally, the slides were then mounted with mounting reagent (DAKO, Hamburg, Germany) and observed by confocal microscopy (LSM 510, Carl Zeiss MicroImaging GmbH, Jena, Germany). Antibodies against GM130 (BD Biosciences, Labware, MA, USA), ERGIC53 (BD Biosciences), and Calnexin (Sigma, St Louis, MO, USA) were used to stain Golgi, Golgi–endoplasmic reticulum (ER) intermediate compartment, and ER, respectively, at a 1 : 100 dilution. The images were captured by confocal microscopy. The scatter plot of the images was generated and the colocalisation coefficient was measured by ImageJ (Available at http://rsbweb.nih.gov/ij/; Developed by National Institutes of Health, Bethesda, MD, USA). Pearson's correlation coefficient indicates the degree of colocalisation, with a value close to 1 meaning perfect colocalisation. Thresholded Mander's split colocalisation coefficient provides the proportion of signals from one channel colocalising with another, with a value of zero meaning no colocalisation and a value of one corresponding to perfect colocalisation (Adler and Parmryd, 2010).

## Results

### Generation and validation of antibodies

**Figure 1:**
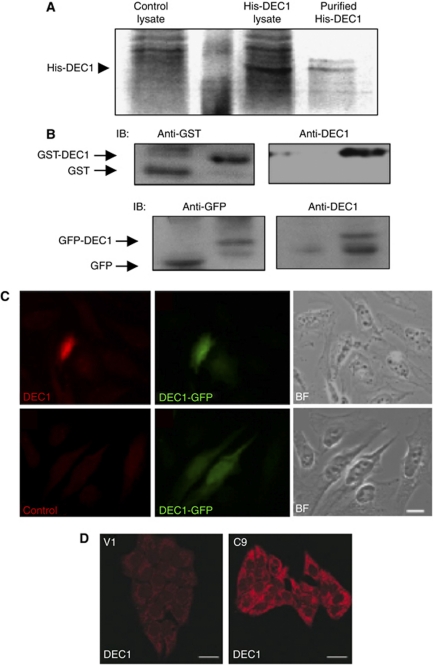
Generation and characterisation of DEC1 antibodies. (**A**) His-tagged *DEC1* proteins were expressed and purified as an antigen to immunise rabbits. (**B**) Upper panel: *DEC1* antibody specifically recognises recombinant GST–*DEC1* fusion proteins, but not GST proteins. Lower panel: the antibody specifically recognises GFP–DEC1 fusion protein, but not GFP. (**C**) In immunostaining, DEC1 antibody specifically recognises GFP–*DEC1* transiently transfected HeLa cells. Nonspecific IgG was utilised as a control. BF, bright field. (**D**) By immunostaining using DEC1 antibodies, higher expression of DEC1 is detected in stable transfectant (C9) than the vector-alone control (V1).

**Figure 2:**
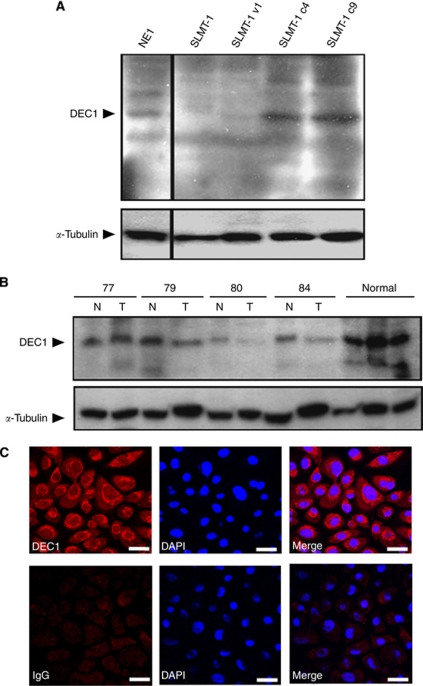
Endogenous DEC1 detection in primary tissues and cell lines. (**A**) Endogenous DEC1 expression in the immortalised epithelial cell line, NE1, and exogenous DEC1 protein in DEC1 stable transfectants (SLMT-1 c4 and c9) were detected by DEC1 antibodies. *α*-Tubulin serves as control for equal loading. (**B**) Downregulation of DEC1 is detected in tumour tissues compared with normal tissues and non-cancer normal individuals. ‘T’, tumour; ‘N’, normal-appearing oesophageal mucosa. (**C**) Upper panel: in NE1, immunostaining shows DEC1 localised in both nucleus and cytoplasm. A vesicular pattern was observed at the perinuclear region. Lower panel: immunostaining of NE1 with IgG primary antibody as a control. ‘DAPI’, nuclear stain (scale bar, 20 *μ*m).

To further our study of DEC1 at the protein level, recombinant His-tagged DEC1 was purified, emulsified in adjuvant, and then used to immunise rabbits (Figure 1). The polyclonal DEC1 antibody can specifically detect GST-tagged DEC1, but not the GST protein (Figure 1B). The DEC1 antibody specifically recognises the mammalian-expressed GFP–DEC1 fusion protein, but not GFP (Figure 1B). By immunostaining using DEC1 antibody, *DEC1–GFP* transiently transfected HeLa cells can be detected (Figure 1C). In addition, ectopic expression of DEC1 can be detected in the *DEC1* stable transfectant (C9) (Figure 1D). All experiments validated the epitope-affinity and specificity of the DEC1 antibody for western blot and immunostaining purposes. Thereafter, DEC1 antibodies were utilised to detect DEC1 protein in cell lines and tissues. Western blot analysis detected expression of both exogenous and endogenous DEC1 in *DEC1* stable transfectants (SLMT-1 c4 and c9) and the immortalised oesophageal epithelial cell line, NE1 (Figure 2A). Loss of DEC1 was observed in oesophageal SCC tumour tissues compared with corresponding normal counterparts (Figure 2B). High expression of DEC1 was also detected in non-cancer normal individuals. Notably, the expression of DEC1 could also be detected by immunostaining. The DEC1 protein locates to both the cytoplasm and nucleus in NE1 and *DEC1* stable transfectants (Figures 1C and 2C).

### DEC1 expression is associated with lymph node metastasis, early onset of oesophageal SCC, and familial oesophageal SCC development

**Figure 3:**
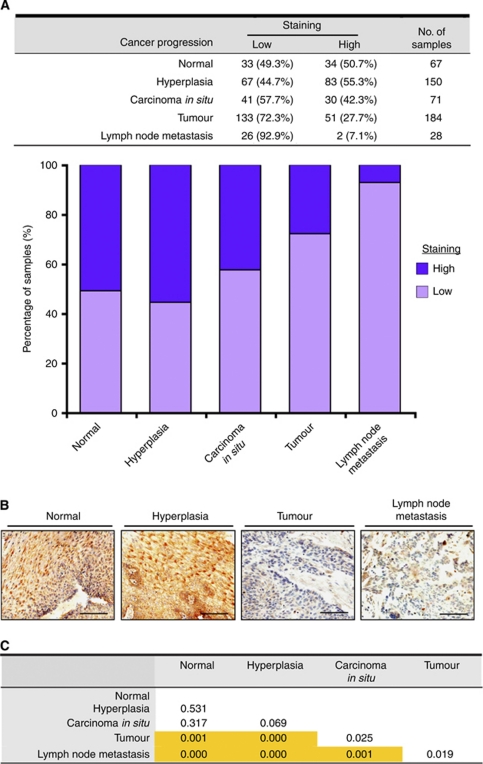
Evaluation of clinical significance of DEC1 by TMA. (**A**) A trend test was performed to correlate DEC1 staining in different levels of cancer progression. The expression of DEC1 continuously decreased from normal/hyperplasia, to carcinoma *in situ*, to tumour, and to lymph node metastasis (Gamma test, −0.44, *P*<0.001). ‘Normal’ includes oesophageal tissues from non-oesophageal SCC patients and normal-appearing oesophageal epithelium from oesophageal SCC patients. (**B**) Representative images in TMA study. High expression of DEC1 in normal and hyperplastic tissues and low expression of DEC1 in tumour and lymph node metastatic tissues are shown. Normal: tissue from non-cancer normal individuals. (Scale bar, 100 *μ*m). (**C**) Chi-square analysis was performed to compare the staining of DEC1 between different levels of cancer progression. A *P*-value <0.005 was regarded as a significant difference after Bonferroni adjustment to control for type I error and is highlighted and bolded.

Tissue microarray analysis was performed using normal epithelium from 32 non-oesophageal SCC patients and tissues from 196 oesophageal SCC patients. First and foremost, the trend test was performed using Gamma test. A value of −0.404 (*P*-value <0.001), indicated a significant trend to show that the cancer progression is negatively correlated with DEC1 expression. Later cancer stages were correlated with lower DEC1 expression. The percentage of high DEC1 staining in hyperplasia is higher than that in normal, but the difference is not statistically significant. Figure 3A shows that expression of DEC1 was attenuated from normal/hyperplasia, to carcinoma *in situ*, to tumour, and to lymph node metastasis progression. Thereafter, a *χ*^2^ test comparing portion of high/low DEC1 staining in all levels of cancer progression indicated that significant difference of DEC1 staining exists between at least two levels of cancer progression (*P*-value <0.001). In order to identify which two levels of cancer progression contained significant differences of DEC1 staining, *χ*^2^ tests were performed to compare DEC1 staining between different levels of cancer progression two by two (i.e., normal *vs* hyperplasia, normal *vs* tumour, etc.). Expression of DEC1 was significantly abated in primary tumours compared with tissues of the normal oesophagus, hyperplasia, and carcinoma *in situ* (*P*-value⩽0.001) (Figure 3C). This is consistent with our previous *in vivo* and *in vitro* functional studies identifying DEC1 as a tumour suppressor of oesophageal SCC (Yang *et al*, 2005; Leung *et al*, 2008). Intriguingly, expression of DEC1 was significantly abrogated in tissues of lymph node metastasis compared with tissues of normal oesophagus and hyperplasia (*P*-value⩽0.001) (Figure 3C), suggesting a clinical association of DEC1 expression with dissemination of oesophageal SCC.

Interestingly, when specimens were analysed according to age groups, the reduced DEC1 expression levels observed in tumours compared with hyperplastic tissues is significant only for younger (30–44 years) and middle-aged (45–69) patients, but not for older (70–79 years) patients (Table 2). The loss of DEC1 expression in younger patients is more significant than in middle-aged ones (*P*=0.001 for younger patients and *P*=0.006 for middle-aged patients). These results suggest DEC1 may have a critical role in early onset of oesophageal SCC development. To determine whether loss of DEC1 is involved in familial oesophageal SCC development, we compared DEC1 expression in oesophageal SCC patients with and without a FH of oesophageal SCC. Significantly lower expression of DEC1 was observed in both hyperplastic and tumour tissues of oesophageal cancer FH+ patients, as compared with oesophageal cancer FH– patients (*P*=0.002 for hyperplasia; *P*=0.006 for tumour) (Table 2). Taken together, the TMA analysis reveals an essential role of DEC1 in not only early onset of oesophageal SCC malignancies, but also familial oesophageal SCC development.

### Subcellular localisation of DEC1

**Figure 4:**
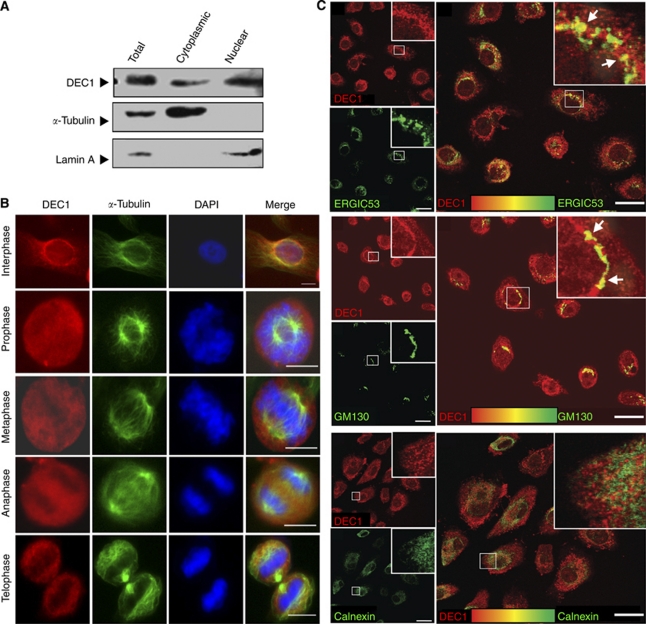
Identifying the localisation of DEC1. (**A**) Subcellular fractionation preparations from NE1 lysates show DEC1 in both cytoplasmic and nuclear fractions. *α*-Tubulin serves as a control for the cytoplasmic fraction. Laminin-A serves as a control for the nuclear fraction. (**B**) Localisation pattern of DEC1 was analysed by immunostaining during cell division by DEC1 antibody. DAPI stain for DNA. (Scale bar, 10 *μ*m) (**C**) Colocalisation of DEC1 with different organelle markers analysed by confocal microscopy. Immunostaining was performed using the immortalised oesophageal epithelial cell line, NE1, with the ERGIC, GM130, Calnexin, and DEC1 antibodies. Upper panel: immunostaining with ERGIC and DEC1 antibodies. ERGIC is a marker for the ER-Golgi intermediate compartment. Colocalisation of *DEC1* with ERGIC was observed (arrow). Middle panel: immunostaining with GM130 and DEC1 antibodies. GM130 is a marker for the Golgi. Colocalisation of *DEC1* with GM130 was observed (arrow). Lower panel: immunostaining with Calnexin and DEC1 antibodies. Calnexin is an ER marker. No colocalisation of *DEC1* with Calnexin was observed. Scale bar, 20 *μ*m. Colocalisations (indicated by arrows) are shown under higher magnification and the region shown in the insert was analysed by ImageJ. The Pearson's correlation coefficient of DEC1 with GM130, ERGIC, and Calnexin is 0.671, 0.437, and 0.287, respectively. Thresholded Mander's split colocalisation coefficient and scatterplot are shown in Supplementary Figure 1.

Identifying the subcellular localisation of a protein provides added insight which may be useful in deciphering its function. The protein localisation program PSORT (www.psort.org) predicts that DEC1 is a cytoplasmic and nuclear protein, while pTARGET (bioapps.rit.albany.edu/pTARGET/) predicts the localisation of DEC1 at the Golgi. We confirmed the subcellular location of DEC1 after subcellular fractionation using lysates from NE1. Deleted in oesophageal cancer 1 was detected in both cytoplasmic and nuclear fractions (Figure 4A). Consistent with this result, immunostaining recognised DEC1 in both the cytoplasm and nucleus. A vesicular pattern of DEC1 staining in the cytoplasm was also detected at the perinuclear region of interphase cells (Figure 4B). Although no specific pattern of DEC1 staining was observed in mitosis (Figure 4B), the vesicular cytoplasmic DEC1 appears to localise in the Golgi and Golgi–ER intermediate compartment (Figure 4C). Organelle-specific markers, GM130 for Golgi; Calnexin for ER; and ERGIC53 for the ER–Golgi intermediate compartment, were utilised. Immunostaining with the organelle markers GM130 and ERGIC53 showed a large overlap with DEC1 (Rp=0.671 for GM130 and Rp=0.437 for ERGIC53; Figure 4C). This was not the case for Calnexin and DEC1 staining and colocalisation is not obvious (Rp=0.287; Figure 4C). In summary, DEC1 localises to the cytoplasm and nucleus and the vesicular pattern of DEC1 in the cytoplasm appears to localise at the Golgi and ER–Golgi intermediate compartment.

## Discussion

The 5-year survival rates for oesophageal SCC are 37.1% for primary cancer, 18.5% for regionally spread cancers, and 3.1% for metastatic cancer (Horner *et al*, 2009). This highlights the importance of cancer research in identifying biomarkers for early diagnosis, to improve survival rates of EC patients. In oesophageal SCC, LOH at marker D9S910 on 9q was associated with metastasis (Hu *et al*, 2000). The 9q31 locus shows an early high frequency of LOH even in low-grade dysplasia of oesophageal SCC (Mori *et al*, 1994). In line with these studies, we previously identified a critical 9q33-34 tumour-suppressive region and found that DEC1 is frequently downregulated at the transcriptional level and functionally involved in suppressing oesophageal SCC tumour formation *in vivo* (Nishiwaki *et al*, 2000; Yang *et al*, 2005; Leung *et al*, 2008). Evidence suggests that DEC1 may serve as a good biomarker for detecting oesophageal SCC. Henan is a high-risk oesophageal SCC area, where the oesophageal SCC incidence rate is as high as 121 per 100 000 population (Xibib *et al*, 2003). This TMA study using tissues from Henan patients revealed a trend of increasingly reduced DEC1 expression with progression from normal, to hyperplasia, to carcinoma, to tumour, and to lymph node metastasis. Not only does this study confirm the crucial tumour-suppressive role of DEC1 in oesophageal SCC, but it also provides a novel finding of a clinical association of DEC1 expression with oesophageal SCC malignancies and metastasis. Interestingly, DEC1 expression is abrogated in tumour tissues from younger oesophageal SCC patients, suggesting its potential utility as an oesophageal SCC detection marker for early onset of oesophageal SCC development.

Having a FH of oesophageal cancer is one of the significant risk factors for oesophageal SCC. This is also associated with several clinical features, including higher prevalence rate of double primary oesophageal SCC and having a worse prognosis than for sporadic cases (Wen *et al*, 2009). Socioeconomic status, living environment, and dietary habits are associated with familial clustering of EC (De Groot, 2006). Not much is known regarding the molecular alterations underlying familial oesophageal SCC, although our recent genome-wide association study has now identified susceptibility loci at PLCE1 and C20orf53 (Wang *et al*, 2010). Downregulation of TGF-*β* signalling (SMAD1) is reported in tumour tissues of familial oesophageal SCC patients (Chattopadhyay *et al*, 2009). In China, allelic loss of chromosome 13 regions was significantly found in patients with a positive FH of oesophageal SCC compared with negative history (Hu *et al*, 2003). Oesophageal SCC family members show aberrant methylation on the promoter of *p16* (Abbaszadegan *et al*, 2005). Here, we document one more molecular alteration associated with this risk factor. Decreased expression of DEC1 in tissues of oesophageal SCC patients is significantly correlated with oesophageal SCC FH status. The statistically significant lower DEC1 staining observed in the FH+ hyperplasia *vs* that in the FH– hyperplasia suggests that loss or reduced DEC1 expression appears to be an early event in ESCC development in FH+ patients. Further study with larger sample sizes is needed for substantiation of the current result. The mechanistic explanation for this observation warrants further investigation.

Three independent *in silico* protein analysis programs, ROSETTA (http://boinc.bakerlab.org/rosetta/), SMART (http://smart.embl-heidelberg.de/), and DisEMBL 1.5 (http://dis.embl.de/) identified intrinsic disorder regions at around 10 residues at the *N*-terminus and the *C*-terminus of DEC1. Intrinsic disorder regions frequently act as sites of posttranslational modifications and are common among cell signalling and cancer-associated proteins (Iakoucheva *et al*, 2002). Its localisation in the cytoplasm and nuclear compartments, as observed in immunostaining and subcellular fractionation experiments, is consistent with its possible role in cellular signalling and signal transduction.

Not only does the Golgi apparatus have a well-characterised function in the secretory pathway, but it also controls centrosome organisation, cell cycle progression, cell polarisation, and cell migration. The Golgi re-orientation towards invadopodia is important for directing cell invasion (Buccione *et al*, 2009). Interestingly, the DEC1 antibody detects vesicular DEC1 located at the Golgi and ER–Golgi intermediate compartments. DEC1 colocalises with GM130, a *cis*-Golgi protein regulating cell migration (Preisinger *et al*, 2004). Indeed, stable expression of *DEC1* in oesophageal SCC cell lines upregulates *dual-specificity phosphatase 6* (Leung *et al*, 2008), a tumour- and cell invasion- suppressor gene that is associated with patient survival in oesophageal SCC (Wong *et al*, 2011). Further investigations are required to elucidate the molecular mechanism of DEC1 in oesophageal SCC.

Taken together, this TMA study reveals the important clinical relevance of DEC1 in lymph node metastatic oesophageal SCC, in early onset oesophageal SCC and familial oesophageal SCC development, further solidifying the crucial role of DEC1 in oesophageal SCC malignancies. This finding adds a novel candidate to the current repertoire of oesophageal SCC diagnostic markers. Moreover, these studies on the subcellular localisation of DEC1 show that it localises to both the cytoplasm and nucleus. Cytoplasmic vesicular DEC1 proteins appear to localise to the Golgi and ER–Golgi intermediate compartment, providing a pivotal clue for further study into the detailed molecular mechanism of DEC1 in oesophageal SCC development.

## Figures and Tables

**Table 1:** Summary of TMA clinico–pathological information for oesophageal SCC patients

	**Number of cases**	**Valid percentage (%)**
*Gender*		
Male	96	63.6
Female	55	36.4
Total	151	
		
*Age*		
⩽44	30	19.9
45–69	86	57.0
⩾70	35	23.2
Total	151	
		
*Tissue type* a		
Normal	32	6.4
Matched normal^b^	35	7.0
Hyperplasia	150	30.0
Carcinoma *in situ*	71	14.2
Tumour	184	36.8
Lymph node metastasis	28	5.6
Total	500	
		
*Stage*		
I–IIa/b	98	66.7
III–IV	49	33.3
Total	147	
		
*Family history* c		
Positive	25	50.0
Negative	25	50.0
Total	50	

Abbreviations: SCC=squamous cell carcinoma; TMA=tissue microarray.

a Not all of the oesophageal SCC patients had all tissue types available. Only 142 patients had matched hyperplastic and tumour tissues.

b Matched normal: normal-appearing oesophageal epithelium from oesophageal SCC patients.

c Data of oesophageal SCC family history are only available in 50 oesophageal SCC patients.

In this TMA study, 32 cases of non-oesophageal SCC normal individuals were also included.

**Table 2:**
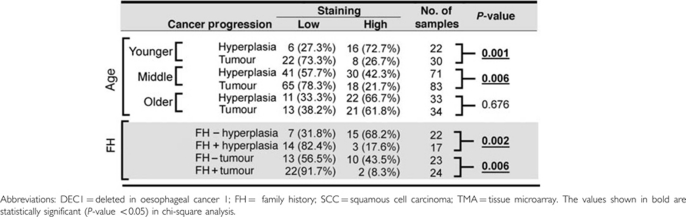
Evaluation of clinical significance of DEC1 in TMA analysis according to patient age and oesophageal SCC familial history
