# Supplementary material for: Abrogated expression of DEC1 during oesophageal squamous cell carcinoma progression is age- and family history-related and significantly associated with lymph node metastasis
Source: Br J Cancer. 2011 Feb 15;104(5):841–9. doi: 10.1038/bjc.2011.25 (PMC3048215; doi:10.1038/bjc.2011.25)

**Supplementary Figure 1** Analysis of co-localisation of DEC1 and organelle markers. Scatter plots are for DEC1 and ERGIC53 (**A**), DEC1 and GM130 (**B**), and DEC1 and Calnexin (**C**). Summary of co-localisation coefficients of DEC1 with different organelle markers are shown in the Table at the bottom. Pearson’s R is Pearson’s correlation co-efficient and M is Mander’s co-localisation coefficients.


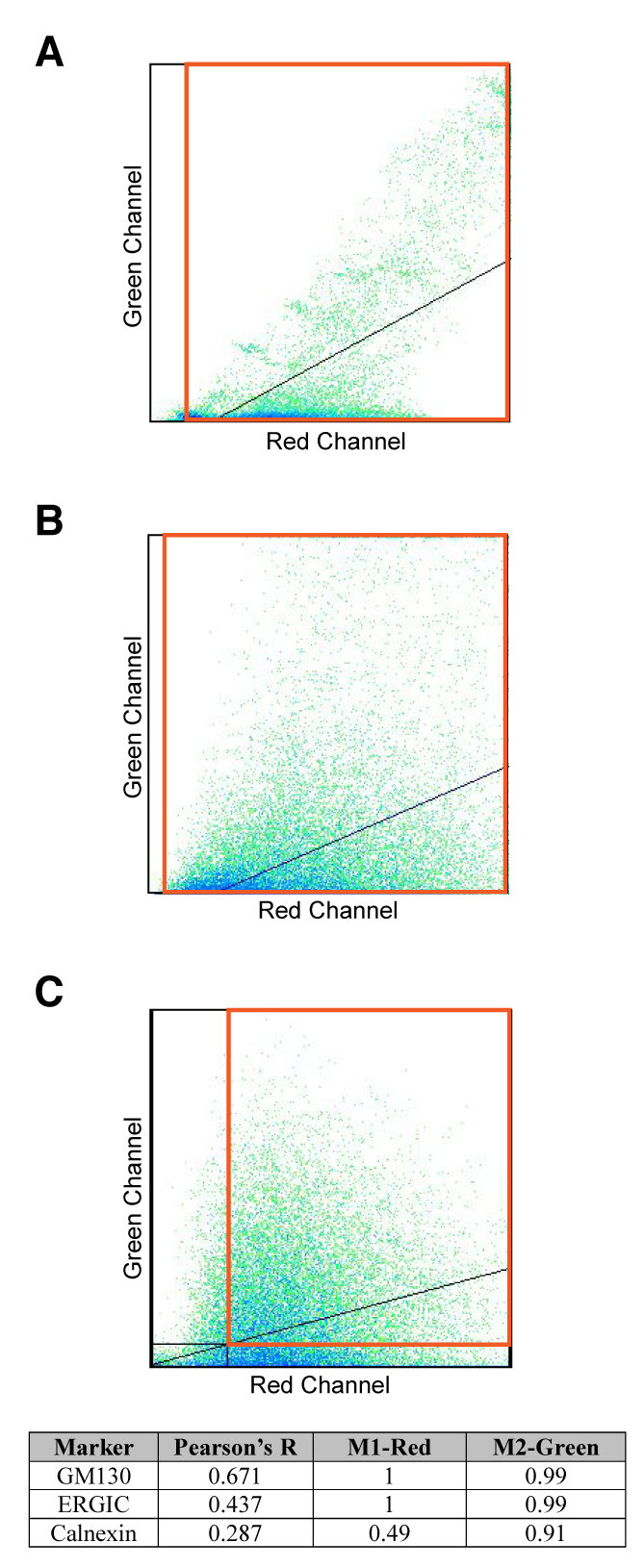

Supplement: Supplementary Figure 1 [file bjc201125x1.doc]
